# Editorial: Oxidative stress and distinct cell death

**DOI:** 10.3389/fphys.2023.1292044

**Published:** 2023-09-21

**Authors:** Yanqing Liu, Quanlu Duan, Liping Peng

**Affiliations:** ^1^ Herbert Irving Comprehensive Cancer Center, Columbia University, New York, NY, United States; ^2^ Division of Cardiology, Department Internal Medicine, Tongji Hospital, Tongji Medical College, Huazhong University of Science and Technology, Wuhan, China; ^3^ Department of Respiratory Medicine, The First Hospital of Jilin University, Changchun, China

**Keywords:** oxidative stress, ferroptosis, apoptosis, cancer, pulmonary fibrosis, rheumatoid arthritis

Programmed cell death is a predefined collective suicide activity of cells in multicellular organisms. It comprises apoptosis, autophagy, ferroptosis, pyroptosis, and other forms of cell death (Galluzzi et al., 2018). It is essential for organism evolution, homeostasis, and the development of our systems. Aberrant regulation in this process is linked to a variety of illnesses, including immunological diseases and developmental abnormalities, neurological diseases, and cancer, suggesting that programmed cell death plays a significant role in physiology and pathophysiology and could be a potential target for therapies in many conditions.

Oxidative stress (OS) is driven by a condition where our bodies’ oxidative and antioxidation systems are out of balance, causing various human diseases, such as aging, carcinogenesis, and degenerative illnesses. Numerous studies have found that oxidative stress plays a pivotal role in the pathogenesis of human disease by promoting cell death, including apoptosis, necroptosis, pyroptosis, ferroptosis, and oxeiptosis, or by disrupting pro-survival signals like autophagy and unfolded protein response (Chio and Tuveson, 2017). Therefore, lowering oxidative stress could shed light on the treatment of these diseases by maintaining the balance between cell death and cell survival. However, this field has not been fully understood: the role and the mechanisms of oxidative stress in diseases need to be further revealed, the regulatory pathways involved in this process should be further identified, and the translational potential must be investigated (Battistelli et al., 2016).

To collect original research papers as well as review articles that will enhance our understanding of the cellular and molecular pathways during OS and/or distinct cell death, we organized this Research Topic called *Oxidative stress and distinct cell death*. Now this Research Topic has been closed. We incorporated five papers into this Research Topic, including three reviews and two research articles. We would like to briefly introduce these papers below.

Ferroptosis is a newly discovered regulated cell death mode named by Brent Stockwell in 2012 (Dixon et al., 2012). Key regulators including GPX4 and p53 have been identified to regulate ferroptosis (Yang et al., 2014; Liu et al., 2019; Liu and Gu, 2021; Liu and Gu, 2022). Ferroptosis is featured by its molecular mechanism that requires iron-dependent lipid peroxidation, which differentiates it from other cell death types. Reactive oxygen species (ROS) is the key to initiate ferroptosis. To summarize the role of ROS in ferroptotic cell death, Endale et al. contributed a review in this Research Topic. They started from introducing the basic knowledge of ROS in cell. Then they focused on how ROS triggers ferroptosis and its regulation. An interesting part is that they touched the topic about the interplay of ferroptosis with other type of cell death in oxidative environment, because ROS can also induce apoptosis and autophagy. Ferroptosis has been demonstrated to be link with many disorders, such as tumors, neurodegenerative disease, and immune diseases (Stockwell et al., 2020). This fact makes ferroptosis a promising target in treating these diseases. Wang et al. introduced the relationship between ferroptosis and pulmonary fibrosis and how targeting ferroptosis may serve as an effective way to treat pulmonary fibrosis. They first reviewed the mechanism of ferroptosis and then depicted how ferroptosis is involved in pulmonary fibrosis development. Finally, they used a separate section to introduce current natural compounds that can target ferroptosis in treatment of fibrosis. In another review by Long et al., authors aimed to clarify the role of ferroptosis in rheumatoid arthritis. They firstly summarized the fundamental pathology of rheumatoid arthritis and the basic mechanism of ferroptosis, separately. Then they link them together to show how ferroptosis contribute to rheumatoid arthritis development. Lastly, they analyzed the therapeutic potential of rheumatoid arthritis by using ferroptosis-related drugs.

Smoking is long recognized as an important oncogenic factor in lung cancer (Barta et al., 2019). In a two-step Mendelian randomization study, Wang et al. found that one underlying mechanism for smoking-associated lung cancer is the oxidative stress and the following programmed cell death. In addition, the authors also identified that the GSTM1 gene polymorphism is associated with smoking-related lung cancer carcinogenesis. This study provides a good model how big data and bioinformatics can be used to study cell death and cancer (Liu et al., 2022; Liu et al., 2023). In primary liver cancer patients who underwent hepatectomy, Li et al. found that dexmedetomidine treatment could decrease the apoptosis of liver cell by lowering the oxidative stress, which improves the liver function. As a consequence, the number of peripheral immune cells in the liver is also reduced.

Taken together, the five papers included in this Research Topic cover both basic science and clinical relevance about oxidative stress and cell death. We believe that these papers will help readers advance their understanding of this field.
